# When Task Constraints Delimit Movement Strategy: Implications for Isolated Joint Training in Dancers

**DOI:** 10.3389/fspor.2020.00049

**Published:** 2020-05-12

**Authors:** Paige E. Rice, Sophia Nimphius

**Affiliations:** ^1^Centre for Exercise and Sports Science Research, School of Medical and Health Sciences, Edith Cowan University, Joondalup, WA, Australia; ^2^Department of Biological Sciences, Northern Arizona University, Flagstaff, AZ, United States; ^3^Sports Performance Research Institute New Zealand, Auckland University of Technology, Auckland, New Zealand

**Keywords:** SSC, strength, power, dancers, training, ankle

## Abstract

Athletic performance is determined by numerous variables that cannot always be controlled or modified. Due to aesthetic requirements during sports such as dance, body alignment constrains possible movement solutions. Increased power transference around the ankle-joint, coupled with lower hip-joint power, has become a preferential strategy in dancers during leaps and may be considered a dance-specific stretch-shortening cycle (SSC) demand. Newell's theoretical model of interacting constraints includes organismic (or individual), environmental, and task constraints describing the different endogenous and exogenous constraints individuals must overcome for movement and athletic performance. The unique task constraints imposed during dance will be used as a model to justify an isolated joint, single-targeted block progression training to improve physical capacity within the context of motor behavior to enhance dance-specific SSC performance. The suggested ankle-specific block progression consists of isometrics, dynamic constant external resistance, accentuated eccentrics, and plyometrics. Such programming tactics intend to collectively induce tendon remodeling, muscle hypertrophy, greater maximal strength, improved rate of force development, increased motor unit firing rates, and enhanced dynamic movement performance. The current perspective provides a dualistic approach and justification (physiological and motor behavioral) for specific strength and conditioning programming strategies. We propose implementation of a single-targeted block progression program, inspired by Newell's theoretical model of interacting constraints, may elicit positive training adaptations in a directed manner in this population. The application of Newell's theoretical model in the context of a strength and conditioning supports development of musculoskeletal properties and control and is conceptually applicable to a range of athletes.

## Introduction

Traditional team sports such as soccer, basketball, volleyball, and baseball involve running, jumping, throwing, and kicking tasks that are afforded numerous degrees of freedom through multiple joints. However, due to rules, judging criteria, or specific joint restrictions, other sports require the same movements to be performed with varying constraints that reduce the degrees of freedom available to solve the same motor task. For example, Paralympic athletes must perform tasks with various structural organismic constraints that result in unique compensatory adaptations of the body or movement strategy. Other situations where environmental constraints are prevalent result in limited or constrained movement available to perform the activity. Water polo players and rowers are restricted by environmental constraints or an additional apparatus that reduce the degrees of freedom available to perform throwing or rowing, respectively. The specific task requirements of other sports (e.g., dancing, figure skating, gymnastics, and diving) require aesthetic body positions that delimit movement solutions due to the constraints of maintaining body alignment during performance tasks. Newell's theoretical model of interacting constraints: organismic (individual), environmental, and task (Newell et al., 1989; Glazier and Davids, 2009), provides a critical framework when training to improve athletic performance.

**Figure 1:**
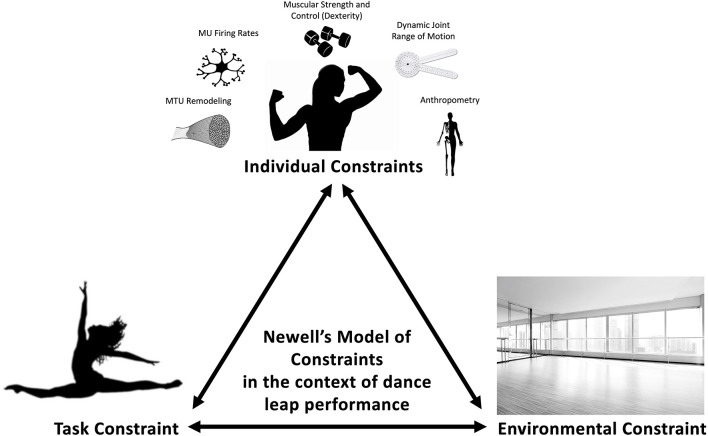
Provides examples of modifiable individual constraints that may contribute to dance performance such as strength, dexterity, range of motion, motor unit firing rate, and MTU remodeling and non-modifiable factors such as skeletal anthropometry. The task constraint of leaps during dance performance requires the torso to remain upright for aesthetics. Finally, the type of flooring, which can interact with either individual or task constraints, within dance can be considered an environmental constraint.

This paper will focus on the implications of modifying organismic constraints (e.g., muscle-tendon unit (MTU) properties, maximal strength, neuromuscular power) of a specific joint as a solution for enhancing performance when task constraints delimit the number or extent to which joints can contribute to perform a task (Figure 1). The unique constraints in dance will serve as a model to justify an isolated joint, single-targeted block progression training to improve physical capacity in the context of motor behavior to enhance dance-specific stretch-shortening cycle (SSC) performance (via increased height or distance leaped). Indirectly, the authors also hypothesize that increases in jumping and leaping from improvements in musculoskeletal properties and control will also reduce the risk of injury from repetition of these movements. In this model, furthering the capabilities of an already highly adapted joint, in contrast to the conventional model of training a “weakness” to enhance performance (Newton and Dugan, 2002), is proposed to be uniquely beneficial. Such a perspective is alluded to from the work of Bobbert and Van Soest (1994), where they pose the question “which of these factors can be changed?” in reference to changing the properties or control of the musculoskeletal system. The acknowledgment of constraints helps direct the effectiveness of a joint, or lack thereof. For example, improvement of a joint that cannot contribute to movement (control) will fail to improve performance despite an improvement in musculoskeletal properties.

During leap propulsion, dancers perform ankle-dominant movement patterns, mainly in styles such as jazz, contemporary, and ballet (Ravn et al., 1999; Jarvis and Kulig, 2016). During these leaps, task constraints stem from the aesthetic requirement of keeping an upright torso. As a result, power transference around the ankle-joint, coupled with lower hip-joint power, has become a preferential movement strategy in dancers during leaps and may be considered a dance-specific SSC demand. Training to enhance the organismic capabilities surrounding the ankle through enhanced MTU properties and neuromuscular capabilities should extend the affordances of a dancer to improve SSC performance even when movement solutions are constrained. MTU properties (e.g., tissue length, pennation angle, stiffness) and force production capability direct how *additional* chronic training may induce physiological and neuromechanical adaptations for improved leap performance. The purpose of this perspective is to provide a theoretical framework that considers physiological adaptations with a motor behavioral lens (e.g., considers motor control and skill acquisition), but specifically applies Newell's theoretical model of interacting constraints to dance-specific SSC actions.

This paper will initially address the demands of constrained movement within dance, followed by the adaptations often elicited in response to such constraints within the MTU. Subsequently, a description for the use of a joint-specific, single-targeted block progression training, including maximal isometric, dynamic constant external resistance (DCER) considered traditional resistance training, accentuated eccentric, and plyometric blocks will be justified. Lastly, the discussion will consider the intent of each training phase relative to the stages of motor learning presented by Newell and how they may guide the acquisition of improved movement coordination and control (Newell, 1985) in conjunction with the physiological intent of each training phase.

## Stretch-Shortening Cycle Performance During Constrained Leaps

In athletes, external mechanical power during the countermovement jump (CMJ) is a surrogate measure of lower limb muscle power (Markovic et al., 2004). As mentioned, the aesthetic task constraints that dancers experience during dance-specific SSC movements generally require the shoulders to remain directly over the hips. In turn, this places a higher demand on the distal segments and joints to generate torque for center of mass (COM) displacement during leaps and jumps. When comparing CMJ performance of dancers and physically active controls, one might expect that dancers would jump higher (Harley et al., 2002). However, this is not always the case (Harley et al., 2002), which may be attributed to reduced hip flexion and torque generation (Imura and Iino, 2017). Dance practitioners continue to measure CMJ ability, although it may not mimic dance-specific SSC movements enough to identify SSC ability when performed with dance-specific constraints. When comparing a leap, such as a saut de chat, to a CMJ, aside from being a unilateral vs. bilateral movement, the most significant difference is that the hips and knees contribute far less torque generation in a saut de chat than a CMJ (Bobbert et al., 1986; McErlain-Naylor et al., 2014; Jarvis and Kulig, 2016). In a study by Jarvis and Kulig, the average peak net moment of the ankle-joint was larger than the hip-, knee-, and metatarsophalangeal-joint moments during the propulsion phase of a saut de chat (Jarvis and Kulig, 2016). Further, Ravn and colleagues found that dancers had 2–3 times higher ankle moments in a ballet-specific jump (sobresaut) than a CMJ (Ravn et al., 1999), and the simultaneous strategy employed by dancers elicited higher ankle-joint angular velocity than the other joints during the sobresaut. Subsequent research, therefore, proposed that an isolated ankle-joint SSC (hop) might better differentiate dancers and untrained individuals. Using a sled apparatus to isolate movement to the ankle, dancers hopped higher during a countermovement hop and drop hops from various heights than untrained individuals (Rice et al., 2018). Additionally, ensemble force- and power-time curves suggested that dancers consistently produced greater relative muscle force and power during the propulsion phase of all the hopping tasks than untrained individuals. Therefore, when constrained movement is considered in the assessment of SSC ability, the difference in performance indicates that dancers' movement strategy is unique compared to most athletes or untrained individuals. More critically, this may explain the divergent findings when using an unconstrained CMJ vs. constrained dance-specific leaps for comparison and justify the importance of considering such constraints for training interventions.

## MTU Properties and Neuromuscular Capability Through Various Ranges of Motion

From a morphological perspective, previous findings have demonstrated that dancers have significantly greater lower leg muscle cross-sectional area (CSA) than that of untrained controls (Rice et al., 2018). We contend that training exposure and habitual stretching are the two main possible explanations for such differences. The repetition of hyper-dorsiflexion and plantarflexion movements that dancers perform may cause the lower leg to undergo hypertrophic cellular signaling via detected contractile stress. The differential myogenic signaling during eccentric muscle actions (in comparison to concentric) likely stimulates higher protein synthesis through the detection of mechanical stress within the sarcomere (Franchi et al., 2017). Specifically, recent evidence suggests that the strain imposed on the Z-disc during eccentric muscle actions activates hypertrophic signaling via muscle LIM protein, titin Z1Z2 domains, and telethonin complexes (Kruger and Kotter, 2016). Mechanosensing properties have also been observed at the M-line's titin-kinase domain, which may partially be responsible for such upregulation of muscle gene expression through inhibition of the muscle ring finger proteins (Linke and Kruger, 2010; Kruger and Kotter, 2016). Albeit much debated, chronic stretching is a proposed stimulus for increased sarcomeres in series, sarcomere CSA, and longer fascicle lengths (Coutinho et al., 2004; Moltubakk et al., 2018), regulated through the abovementioned cellular mechanisms. Such justification may explain why professional ballet dancers possess significantly longer medial gastrocnemius fascicle lengths and greater muscle thickness than physically active controls (Moltubakk et al., 2018).

Certain sport-specific movements that involve substantial repetition may induce alterations in muscle force-length and force-velocity properties. The repetitive passive and active hyper-plantarflexion most dancers experience daily around the ankle-joint may transcend a shorter operative MTU length for maximal force transmission during relevé or toe-off before a leap, shifting the archetypal force-length curve to the left (Frasson et al., 2008; Moltubakk et al., 2018). However, dancers also seem to move through hyper-dorsiflexed positions during grand plié or landing from a leap. Dance has been referred to as a unique form of eccentric exercise for movements involving extreme ranges of motion (ROM) and unilateral force absorption (Paschalis et al., 2012). Eccentric muscle actions may eventually damage the sarcomeres on the descending limb of the force-length curve and induce a (right) shift in the optimum muscle length at which peak torque occurs (Gregory et al., 2007). It has been speculated that this is due to greater muscle compliance evoked by repeated eccentric muscle actions (Gregory et al., 2007). Over time, such ankle excursion might cause physiological adaptations that optimize force output and MTU interaction at new muscle lengths. In a comparative study, dancers reached higher isometric plantarflexion peak torque values than volleyball players at all ankle angles measured, and it was evident that the dancers favored shorter plantar flexor lengths (Frasson et al., 2008). However, no differences existed between groups for the reported peak isokinetic torque. In another study, dancers had significantly greater isokinetic torque in more plantarflexed ankle positions than controls (Moltubakk et al., 2018), suggesting that the study mentioned above may have neglected existing differences by only analyzing peak torque. While the force-length and force-velocity profiles of dancers remain undetermined, it is evident that dancers require force generation over a range of muscle lengths and angular velocities (adagio vs. allegro) about the ankle-joint.

The type of training dancers undergo from a young age would be expected to influence tendinous tissue properties as well. For example, professional ballet dancers possess significantly longer and more compliant Achilles tendons than controls (Moltubakk et al., 2018). Further, dancers have a significantly higher average resultant musculo-articular stiffness than untrained controls when utilizing a free-oscillation technique to measure triceps surae complex stiffness (Rice et al., 2017). Such tendinous adaptations may enhance the MVIP rate of force development (RFD), as this has been correlated to musulo-articular stiffness. Previous work has demonstrated that fascicle and tendinous tissue shortening velocity simultaneously increase with angular velocity during isokinetic contractions (Hauraix et al., 2015). Greater fascicle-shortening velocity also significantly correlated to longer fascicle lengths. Thus, the longer fascicles in dancers may induce greater muscle fascicle-shortening and allow for a more rapid strain imposed on tendinous tissues, increasing triceps surae complex stiffness during SSC actions. The SSC involves the lengthening of the entire MTU, which stores elastic strain energy that is subsequently utilized upon shortening during propulsion to enhance jumping or leaping performance. As the MTU has commonly been modeled as a damped mass-spring, stiffer musculature, and tendinous tissues with greater elongation may facilitate greater energy storage and return during SSC actions (Lichtwark and Wilson, 2005). Dancers have a unique ability to adapt to different flooring and gear joint stiffness levels accordingly, perhaps due to leaping demands (Hackney et al., 2011). Ultimately, it is essential to individually optimize viscoelastic properties particularly surrounding the ankle in dancers through training. Understanding the need to enhance lower leg MTU function in dancers directs us toward an ankle-specific block progression including isometrics, DCER, accentuated eccentrics, and plyometrics from a physiological adaptation perspective. However, these blocks are also ordered to enhance the motor learning that is requisite for task constraints in dance (Figure 1).

## Block Progression Training

**Figure 2:**
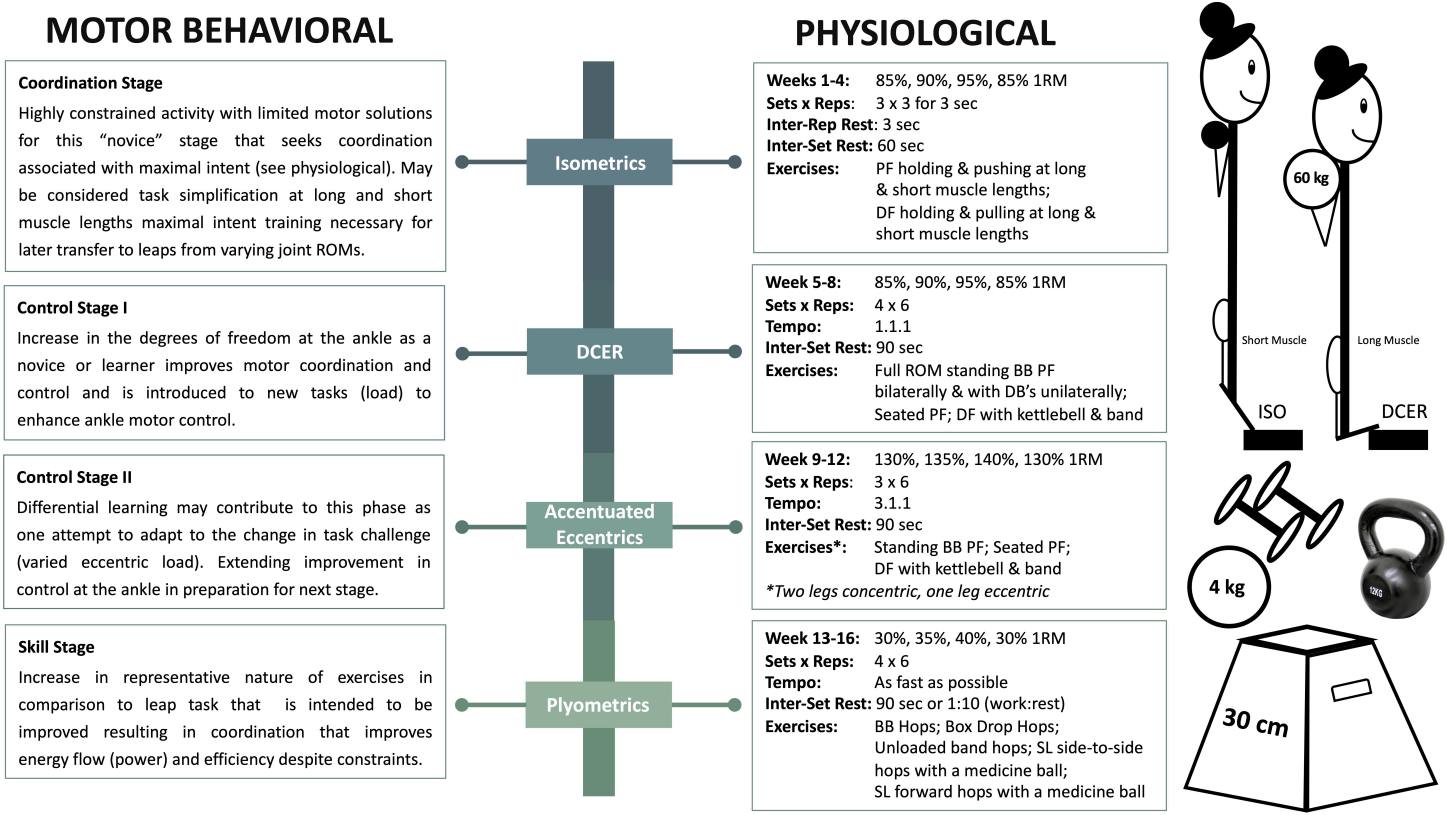
Illustrates an example of a single-targeted block progression training with phase potentiation including intensity, volume, duration, and exercises. ISO, isometrics; DCER, dynamic constant external resistance; PF, plantarflexion; DF, dorsiflexion; ROM, range of motion; BB, barbell; DB, dumbbell; SL, single-leg. The stages of motor learning are described in Newell (1985). It should be noted that the linear nature of the proposed phases could be rearranged from a skill acquisition perspective or combined (differential learning). More research is required to determine if the ordered phases proposed from a physiological perspective are superior to any other ordering of phases. Further, despite dancers being in the “skill stage” in dance specific leaps, the use of the phases proposed with various changes to the task are in themselves differential learning approaches for even these skilled performers.

Single-targeted block progression training with phase potentiation “*aims to develop a single fitness characteristic while maintaining previously developed characteristics”* (Suchomel et al., 2018). Suchomel et al. describe the manifold benefits of such an approach for athletes that must peak in a timely fashion for competitive performance. Through the application of concentrated loads, individuals may realize desired training attributes during successive blocks via phase potentiation. Isometric training, DCER, accentuated eccentrics, and plyometrics are intended to collectively induce tendon remodeling, muscle hypertrophy, maximal strength gains, improved RFD capacity, increased motor unit firing rates, and enhanced dynamic movement performance (Figure 2). In addition to physiological changes to the individual, such adaptations should be considered in the context of motor performance, which must be considered in the context of task constraints. Therefore, we will merge physiological and motor behavior frameworks to justify the proposed isolated-joint, single-targeted block progression. The subsequent blocks are proposed with a focus on constraining the exercises to ankle-joint focused movements (e.g., calf raises, dorsiflexion, and hip constrained jumps such as ankle hops).

Isometric exercise is the intention to push, pull, or hold in a joint position without realization of external movement (Lum and Barbosa, 2019). The lower utilization of ATP during isometric muscle actions in comparison to shortening muscle actions suggests that athletes' time-to-fatigue may increase during training sessions, allowing them to sustain a greater volume of training (Newham et al., 1995). A recent review highlighted that isometric training has shown to increase strength and dynamic performance; however, exercise prescription is a critical component for improvement (Lum and Barbosa, 2019). For example, isometric training at different quadriceps muscle lengths (Kubo et al., 2006) and long- vs. short-duration (Kubo et al., 2001) influences the adaptations to tendon stiffness and maximal torque levels. Isometric training at longer muscle lengths for longer durations has shown to result in significant increases in tendon stiffness and maximal strength levels across a spectrum of joint-angles. Collectively, these results demonstrate that joint-angle and time under tension appreciably impact the stimulus introduced to the MTU for mechanical adaptation. Maximal isometric efforts may have further implications from a motor learning perspective in the current and subsequent training blocks (Behm and Sale, 1993) as outlined in Figure 2. The proposed stages of learning within each phase are associated with the constraints, intent, or degrees of freedom available based on the exercises of each phase. In applying this concept to dancers, a block of performing maximal intent isometric contractions in a more dorsiflexed position (longer muscle length) may enhance RFD as a result of mechanical and control-based musculoskeletal adaptations, which is critical for increasing impulse before the take-off of a leap for maximal COM displacement (Kirby et al., 2011). Therefore, the first block of ankle-focused maximal isometrics is intended to set the foundation for continued realization in successive blocks (Epro et al., 2018), transitioning into exercises that include the entire joint ROM.

DCER involving both lengthening and shortening MTU actions through a full ROM has been studied from several aspects of neuroplasticity and neuromechanical development (Aagaard et al., 2002). DCER at higher intensities or loads has been shown to elicit an increase in type IIA MHC expression (Andersen and Aagaard, 2000), type II muscle fiber CSA (Aagaard et al., 2001), and peak force and power of all fiber types (Widrick et al., 2002). Other combined training programs that included DCER and loaded ballistic exercises have seen improvements in RFD, maximal strength, and jumping performance (Kyrolainen et al., 2005), interestingly with no apparent changes in titin isoform expression. However, mechanical tests of myofibrils or myofibers may better demonstrate functional adaptations to titin rather than gel electrophoresis, which detects only very large changes. In this model, DCER will allow initial intent and physiological adaptations to continue developing while furthering increases in type II MHC expression of the plantarflexor muscles, muscle fiber CSA, MVIP peak force, and RFD to enhance leap performance. Load, velocity, and time under tension of exercises may be manipulated to provide greater specificity in the projected adaptation. Augmentation of motor control to maximize intent through coordination at angle relevant positions of the ankle and initial physiological adaptations from the isometric and DCER blocks will also prepare the athlete for the most mechanically demanding block, accentuated eccentric loading.

Accentuated eccentric loading (AEL) is considered similar to DCER training; however, a supramaximal load during the eccentric phase is prescribed for maximal strength and power realization. Some known physiological effects of AEL are increased number of sarcomeres, type II MHC expression, muscle fiber CSA, fascicle length, muscle CSA, tendon stiffness, and bone mass (Foure et al., 2013; Vogt and Hoppeler, 2014; Hessel et al., 2017). Furthermore, AEL, in addition to isometrics, is often prescribed for individuals suffering from Achilles tendinopathy (Maganaris et al., 2017), which is prevalent in dancers. As such, the culmination of the previous blocks and AEL may increase tendon stiffness to withstand higher tensile forces through stimulation of collagen synthesis, and thereby reduce injury risk (Bohm et al., 2015; Maganaris et al., 2017). Practically, AEL exercises are performed using methods including elastic bands, weight releasers, or manual adjustments (e.g., calf raise: two-legged concentric with 80%, single-legged eccentric with 130%). AELs induce higher forces whilst the energy cost is low (Wagle et al., 2017), shown to improve SSC mechanisms and athletic performance (e.g., sprinting, jumping, and throwing) (Aboodarda et al., 2014; Wagle et al., 2017). Therefore, supplementing dance-specific training with AEL may significantly increase muscle fiber properties, tendon remodeling, and eccentric kinetics for enhanced performance and injury prevention (Vogt and Hoppeler, 2014; Franchi et al., 2017; Maganaris et al., 2017). The exaggerated eccentric component during AEL may enhance neural commands and thus, improve motor behavior specifically through enhancing control or motor learning from the differential nature of changing the eccentric load (Bobbert and Van Soest, 1994). Further, conditioning the MTU to sustain higher loads of tensile force and thus, facilitating an enhanced ability to withstand high training loads, may concurrently allow for improvement in skill acquisition through enhanced efficiency and reduce injury risk (Figure 2).

The final block, proposed to actualize enhanced organismic constraints into motor performance, is a plyometric block that returns to loads most representative in task to dance performance for specificity and maximized transference. As such, this may facilitate the “skill phase” of the stages of motor learning (Figure 2). Plyometric exercise is commonly defined as an overloaded eccentric phase, ballistic SSC, beneficial for fine-tuning utilization of stored elastic energy (Malisoux et al., 2006). Plyometrics increase unloaded shortening velocity and absolute peak power of muscle fibers (Malisoux et al., 2006), active muscle stiffness (Kubo et al., 2017), and muscle pre-activity and eccentric phase muscle activity, possibly due to the increased demand placed on the muscle to maintain a quazi-isometric state (McBride et al., 2008). Specifically, plyometric training that involves the intention to move as quickly as possible through a SSC may also result in activation of higher threshold motor units for maximal power output (Behm and Sale, 1993). Thus, in-depth analysis of the literature suggests plyometric training causes substantial strength gains coupled with DCER training due to enhanced neuromuscular function and proper time sequencing of training modalities (Saez-Saez de Villarreal et al., 2010). Development of neuromechanical function will subsequently result in modulation of motor unit synchronization, operative force transmission, and maximal execution of SSC movements. Therefore, prescription and practice of plyometrics should specifically address adaptations from ankle-specific exercises to enhance leaping capability. We contend that dancers might benefit from single-targeted block progression of ankle-specific movements distinctly in comparison to “traditional training” for improvements in dance-specific SSC performance and potential for reduced risk of injury. However, we note that such approach may best benefit an athlete after holistic development and as a phased approach as part of the greater annual plan. Such periodization should also be complimented with broader strength and conditioning approaches to reduce monotony and place a balance between performance and risks associated with excessive specialization. Finally, as noted in Figure 2, there is greater understanding and research required from a physiological perspective whether training phases can be introduced in different orders to produce similar performance outcomes. A differential learning perspective for such benefit is discussed in Figure 2.

## Conclusion

The current perspective provides a dualistic approach and justification (physiological and motor behavioral) for specific strength and conditioning programming strategies. Simultaneously, we considered the necessary changes in motor control that may occur with certain training modalities typically programmed to actualize physiological adaptations. The aim of an isolated joint training program may differ depending on the population's constraint, whether organismic-, environmental-, or task-oriented. Dancers require the ability to sustain large amounts of stretching, muscular contractions, and cyclic, maximal SSC movements. To amplify the strength and power capabilities of these athletes, we took into consideration a spectrum of literature from basic science, motor behavior, and strength and conditioning research. In conclusion, we propose implementation of a single-targeted block progression program with greater context of Newell's theoretical model of interacting constraints, may elicit positive training adaptations in dancers by enhancing musculoskeletal properties and musculoskeletal control. However, the abovementioned adaptations proposed with the single-targeted block progression may be applicable and beneficial to other athletes, but the efficacy of the specific order or progression should be further explored from a motor behavior and physiological perspective.

## Author Contributions

PR and SN contributed to the theoretical development of the perspective. PR and SN further contributed to the drafting and editing of the perspective.

## Conflict of Interest

The authors declare that the research was conducted in the absence of any commercial or financial relationships that could be construed as a potential conflict of interest.
